# Genetic background mutations drive neural circuit hyperconnectivity in a fragile X syndrome model

**DOI:** 10.1186/s12915-020-00817-0

**Published:** 2020-07-30

**Authors:** Tyler Kennedy, David Rinker, Kendal Broadie

**Affiliations:** 1 Department of Biological Sciences, Vanderbilt University and Medical Center, Nashville, TN 37235 USA; 2 Department of Cell and Developmental Biology, Vanderbilt University and Medical Center, Nashville, TN 37235 USA; 3 Vanderbilt Brain Institute, Vanderbilt University and Medical Center, Nashville, TN 37235 USA

**Keywords:** Giant fiber, Fragile X syndrome, Genetic background, Bulk Segregant analysis, *Drosophila*, Neuron, Synaptogenesis, Circuit formation, Projection, Synaptic wiring

## Abstract

**Background:**

Neural circuits are initially assembled during development when neurons synapse with potential partners and later refined as appropriate connections stabilize into mature synapses while inappropriate contacts are eliminated. Disruptions to this synaptogenic process impair connectivity optimization and can cause neurodevelopmental disorders. Intellectual disability (ID) and autism spectrum disorder (ASD) are often characterized by synaptic overgrowth, with the maintenance of immature or inappropriate synapses. Such synaptogenic defects can occur through mutation of a single gene, such as fragile X mental retardation protein (FMRP) loss causing the neurodevelopmental disorder fragile X syndrome (FXS). FXS represents the leading heritable cause of ID and ASD, but many other genes that play roles in ID and ASD have yet to be identified.

**Results:**

In a *Drosophila* FXS disease model, one *dfmr1*^*50M*^ null mutant stock exhibits previously unreported axonal overgrowths at developmental and mature stages in the giant fiber (GF) escape circuit. These excess axon projections contain both chemical and electrical synapse markers, indicating mixed synaptic connections. Extensive analyses show these supernumerary synapses connect known GF circuit neurons, rather than new, inappropriate partners, indicating hyperconnectivity within the circuit. Despite the striking similarities to well-characterized FXS synaptic defects, this new GF circuit hyperconnectivity phenotype is driven by genetic background mutations in this *dfmr1*^*50M*^ stock. Similar GF circuit synaptic overgrowth is not observed in independent *dfmr1* null alleles. Bulked segregant analysis (BSA) was combined with whole genome sequencing (WGS) to identify the quantitative trait loci (QTL) linked to neural circuit hyperconnectivity. The results reveal 8 QTL associated with inappropriate synapse formation and maintenance in the *dfmr1*^*50M*^ mutant background.

**Conclusions:**

Synaptogenesis is a complex, precisely orchestrated neurodevelopmental process with a large cohort of gene products coordinating the connectivity, synaptic strength, and excitatory/inhibitory balance between neuronal partners. This work identifies a number of genetic regions that contain mutations disrupting proper synaptogenesis within a particularly well-mapped neural circuit. These QTL regions contain potential new genes involved in synapse formation and refinement. Given the similarity of the synaptic overgrowth phenotype to known ID and ASD inherited conditions, identifying these genes should increase our understanding of these devastating neurodevelopmental disease states.

## Background

During neural circuit formation, axons and dendrites extend transitory processes that contact potential partners [1–3]. This initial synaptic connectivity is coordinated by a complex array of secreted morphogens, transmembrane receptors, and cytoskeletal regulators [4–6]. Nascent synapses are usually formed in excess, overgrowing both appropriate and inappropriate targets, only to be refined over time through retraction and/or glial pruning, to sculpt the mature synaptic connectivity patterns [7]. With the onset of environmental sensory input, this refinement process continues, mediated by multiple activity-dependent synaptic mechanisms [8]. Genetic disruption of this precise synapse initiation and maturation program causes neurodevelopmental disorders of intellectual and autistic disabilities [9]. The most common heritable state is fragile X syndrome (FXS), a monogenic disorder and key model for studying links between synaptic connectivity and disease [10].

The FXS disease state is caused by genetic loss of mRNA- and channel-binding fragile X mental retardation protein (FMRP [11, 12]). It has been repeatedly confirmed in both mouse and *Drosophila* FXS models that FMRP loss alone can cause circuitry and behavioral defects recapitulating the human condition. Both FXS patients and disease models manifest the hallmark phenotype of excess, immature synapses [13]. This synaptic overgrowth has been well documented in both presynaptic boutons and postsynaptic spines, and studies have also identified overgrown axonal and dendritic branches [14–20]. Many diverse mechanisms drive synaptic overelaboration, including enhanced metabotropic glutamate receptor (mGluR) signaling, elevated microtubule and actin cytoskeleton stabilization, and disrupted synapse pruning [19, 21, 22]. However, in both FXS patients and models, genetic background has a profound impact on the penetrance and severity of synaptic defects [23–25].

In the current study, we employ the well-characterized *Drosophila* FXS disease model to pursue mechanisms of synaptic connectivity defects. Specifically, we focus on the giant fiber (GF) neural circuit due to its large size, well-mapped neurons, and targeted transgenic tools [26, 27]. We sought to model FXS synaptic connectivity defects in this tractable circuit with single-cell resolution to test the numerous proposed disease mechanisms. We focus particularly on the GF interneuron (GFI), a bilaterally symmetric neuron pair, with cell bodies and dendrites in the central brain, and large axons projecting into the thoracic ganglia [28]. The GFI axons use mixed electrical and chemical synapses to connect first with the peripherally synapsing interneuron (PSI) and giant fiber coupled 1–4 (GFC1–4) neurons at the inframedial bridge (IB), and then diverge to form two large bends that synapse onto the tergotrochanteral motor neuron (TTMn) and GFC2–3 neurons [27, 29–31].

We initially set out to test whether the GFI displays synaptic overgrowth using a well-characterized FMRP null allele (*dfmr1*^*50M*^ [21]). Consistent with reports from other circuits, *dfmr1*^*50M*^ displays excess GFI filopodia during synaptogenesis and more GFI mature synapses [20, 21, 32–34]. The excess GFI projections synapse onto GFC2/3, indicating synaptic overgrowth is redundant within the GF circuit, without inappropriate connections. This intra-circuit hyperconnectivity is more weakly manifest in *dfmr1*^*50M*^ heterozygotes, suggesting semi-dominance, and significantly rescued by re-introduction of wildtype FMRP, suggesting a FMRP-specific requirement. However, our studies reveal FMRP loss does not cause the defect, which is instead dependent on background mutations in a *dfmr1*^*50M*^ stock. We therefore used bulked segregant analysis (BSA) with whole genome sequencing (WGS) to identify these mutations [35]. Our results identify 8 loci driving the intra-circuit hyperconnectivity.

## Results

### A FXS disease model exhibits excess GFI axonal projections

**Fig. 1:**
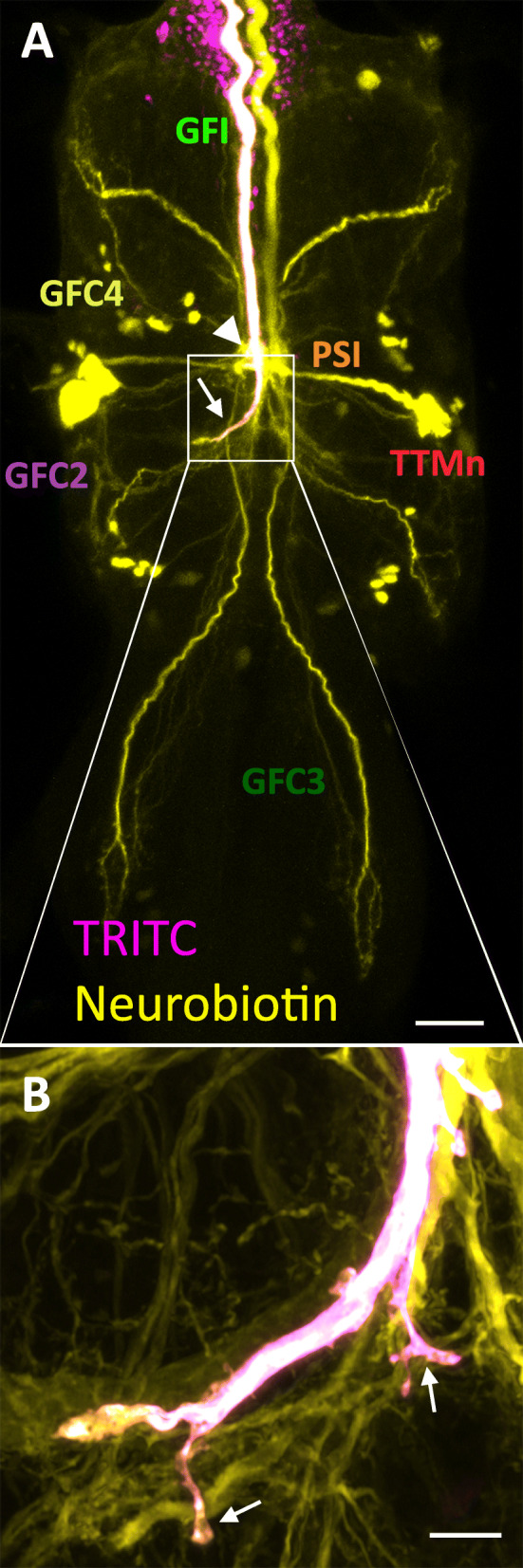
Presynaptic projections from the giant fiber interneuron terminal bend. **a** Co-injection of TRITC-dextran (10 kDa; magenta) and neurobiotin (287 Da; yellow) into a *w*^*1118*^ giant fiber interneuron (GFI) axon labels the neuron and all the gap junction dye-coupled GF circuit neurons. Visible are peripherally synapsing interneuron (PSI), tergotrochanteral motor neuron (TTMn) cell body, and giant fiber coupled (GFC) 1–4 neurons, which were just recently characterized (Kennedy and Broadie 2018). The GFI inframedial bridge (IB, arrowhead) and GFI axonal bends (arrow) located in the second thoracic ganglion segment are the two presynaptic sites. **b** Enlarged image of one GFI axonal bend (see box in **a**) showing the newly identified presynaptic projections (arrows). Scale bars, 25 μm (**a**) and 5 μm (**b**)

The central brain giant fiber interneuron (GFI) can be labeled at single-neuron resolution by injecting TRITC-dextran into the axon in the cervical connective (Fig. 1a; ([36]). Co-injecting with the small gap junction-permeant neurobiotin (NB) tracer labels electrically coupled partners (Fig. 1a [29, 37]). The primary presynaptic sites of the GFI are at the inframedial bridge (IB; Fig. 1a, arrowhead), which synapses with the peripherally synapsing interneuron (PSI) and giant fiber coupled (GFC) neurons 1–4, and the axonal bends (Fig. 1a, arrows), which synapse with the tergotrochanteral motor neuron (TTMn) and GFC2–3 ([29, 30, 38]). In addition, the GFI can be genetically targeted at near single-cell resolution using the 91H05-Gal4 driver, permitting a myriad of GFI transgenic manipulations ([39–41]). Using either GFI-targeted expression of membrane-tethered GFP (mCD8::GFP) or the iontophoretic co-injection of TRITC-dextran and NB tracer dyes, we identified small projections along the distal GFI axonal bend of the *w*^*1118*^ genetic control (Fig. 1b, arrows). These putative synaptic contacts were therefore assayed in our fragile X syndrome (FXS) model, which is characterized by disrupted synapse formation and activity-dependent refinement ([14, 15, 19]).

**Fig. 2:**
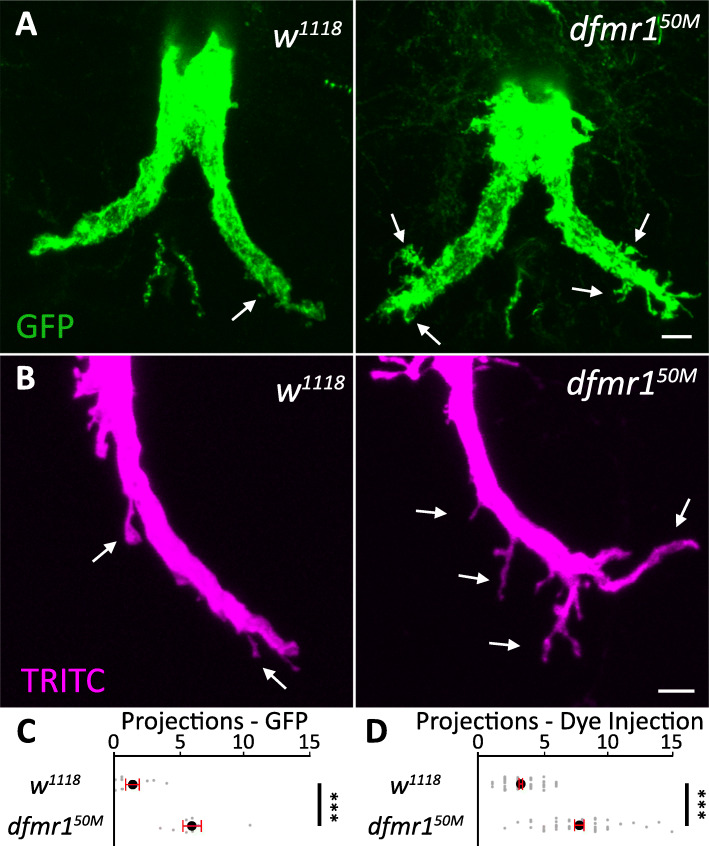
Supernumerary GFI axonal projections in *dfmr1*^*50M*^ null mutants. **a** The giant fiber interneuron (GFI) visualized with 91H05-Gal4 driving the membrane marker UAS-*mcd8*::*gfp* (green) in the *w*^*1118*^ genetic background control (left) and the *dfmr1*^*50M*^ null mutant (right). In the controls, GFI axonal bends have relatively few projections compared to an excess number of overgrown projections in the mutants. Arrows indicate representative axon projections. **b** Iontophoretic TRITC-dextran dye injection (magenta) in *w*^*1118*^ (left) and *dfmr1*^*50M*^ (right) stocks show the same projection phenotype. Scale bars, 5 μm. **c** Quantification of the GFP-labeled axonal projections. Each gray dot represents the average projection number of both bends in one animal. The black dot represents the average, and the red bars represent the standard error of the mean. Sample sizes: *w*^*1118*^ (*n* = 8) and *dfmr1*^*50M*^ (*n* = 8) animals. **d** Quantification of the TRITC-labeled projections. Each gray dot represents an axon bend in one animal. Sample sizes: *w*^*1118*^ (*n* = 62) and *dfmr1*^*50M*^ (*n* = 48)

We first compared genetic background control (*w*^*1118*^) and *dfmr1* null (*dfmr1*^*50M*^) GFIs expressing mCD8::GFP and found a very strong axonal projection phenotype when FMRP is removed (Fig. 2a). In controls, GFI axon bends display only a few projections, whereas *dfmr1* mutants have many projections from the bends, often of substantive size and complexity (Fig. 2a, arrows). Quantification of projections ≥ 2 μm in length shows controls have an average of 1.4 ± 0.5 projections/bend, while *dfmr1* nulls have 6.0 ± 0.7, a significant increase (*p* = 1.6 × 10^−4^, two-tailed unpaired *t* test; Fig. 2c). To ensure these projections are not caused by the Gal4 driver or UAS responder, we next dye-injected GFI axons with TRITC-dextran in *w*^*1118*^ and *dfmr1*^*50M*^ (Fig. 2b). When projections are compared with this labeling strategy, we again find supernumerary processes in *dfmr1*^*50M*^ relative to the *w*^*1118*^ control (Fig. 2b). Quantification shows control GFIs have an average of 3.3 ± 0.2 projections/bend whereas *dfmr1* nulls have 7.8 ± 0.4, again a significant elevation (*p* = 4.7 × 10^−20^, two-tailed unpaired *t* test; Fig. 2d). Together, these findings suggest GFI axonal projections would be ideal to study how FMRP loss affects circuit connectivity in the *Drosophila* FXS model. The projection phenotype is robust and relatively easy to measure, so we sought to characterize the defect more fully before dissecting the molecular mechanism responsible for the overgrowth.

### Increased axonal projections present during early GFI synaptogenesis

**Fig. 3:**
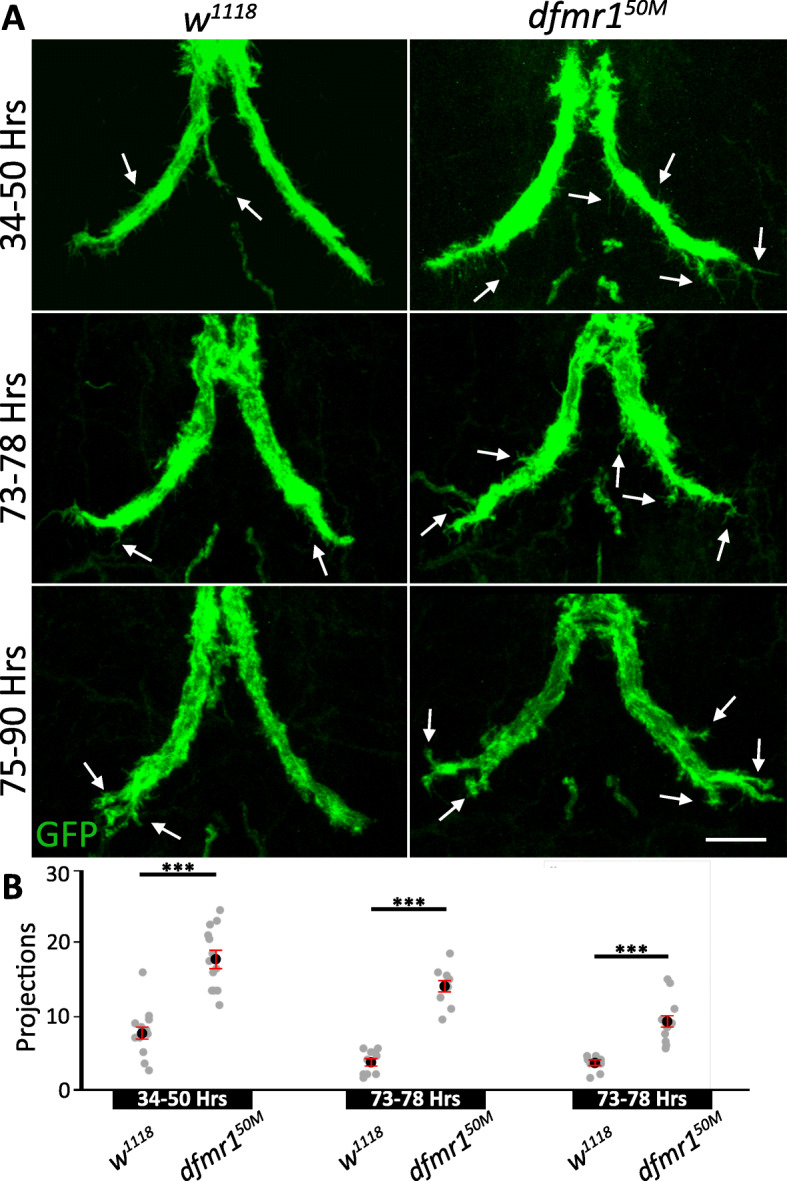
GFI axonal projection overgrowth begins early in synaptogenesis. **a** The giant fiber interneuron (GFI) axonal bend visualized with 91H05-Gal4 driven UAS*-mcd8::gfp* (green) during staged development in background controls (*w*^*1118*^, left) and *dfmr1* null mutants (*dfmr1*^*50M*^, right). The three time points are during (1) initial GFI synaptogenesis at 34–50 h after puparium formation (APF, top), (2) GFI synapse maturation at 73–78 h APF (center), and (3) GFI synapse stabilization at 75–90 h APF (bottom). Arrows indicate representative GFI axonal bend projections. Scale bar, 10 μm. **b** Quantification of the axonal projections at all 3 time points for both genotypes. Each gray dot represents the projection number for an axon bend in one animal. The black dot represents the mean and red bars represent the standard error of the mean. Sample sizes: 34–50 h: *w*^*1118*^, *n* = 12; *dfmr1*^*50M*^, *n* = 13. 73–78 h: *w*^*1118*^, *n* = 10; *dfmr1*^*50M*^, *n* = 10. 75–90 h: *w*^*1118*^, *n* = 12; *dfmr1*^*50M*^, *n* = 12

We first investigated when the overgrown axonal projections develop during GF neural circuit formation. At 25 °C, the GFI reaches its TTMn target at ~ 24 h after puparium formation (APF [30]), at which point synaptogenesis begins. The initial phase of synapse formation then lasts for approximately 1 day (24–48 h APF [30]). In order to examine this synaptogenesis period, we collected animals staged at 34–50 h APF by selecting for the “yellow body” localized between the Malpighian tubules on the dorsal side of the pupae [42]. Using GFI-specific 91H05-Gal4 to drive UAS-*mcd8*::*gfp*, we assayed axonal bend projections during this early time period (Fig. 3a). Both background control (*w*^*1118*^) and *dfmr1* null (*dfmr1*^*50M*^) GFIs exhibit far more extensive projection outgrowth at 34–50 h APF than at maturity, but the mutants show a much greater elevation (Fig. 3a, arrows). These early GFI projections are usually far more slender than the projections at maturity suggesting they are immature filopodial processes searching for synaptic partners [2, 43]. Quantification shows controls have 7.8 ± 1.0 projections/bend whereas *dfmr1* nulls have 17.8 ± 1.2, a highly significant elevation (*p* = 1.3 × 10^−6^, two-tailed unpaired *t* test; Fig. 3b, left). Thus, the FXS model defect is apparent from the early stages of synaptogenesis.

We next assayed later stages in GF circuit development, testing two time points during synaptic maturation (Fig. 3a): 73–78 h APF and 75–90 h APF, identified by gray and black colored pupal wings, respectively. At each time point, *dfmr1* nulls display more GFI axonal projections compared to the matched controls, albeit with a progressive decrease in projection number over time (Fig. 3a, b). Note *w*^*1118*^ controls decrease projection number by 73–78 h APF, but show little decline at 75–90 h APF (Fig. 3a). Quantification supports these observations, showing 73–78 h APF controls have only 3.7 ± 0.5 projections/bend, whereas *dfmr1* nulls have 14.1 ± 0.8 (*p* = 2.7 × 10^−9^, two-tailed unpaired *t* test; Fig. 3b, middle). By 75–90 h APF, controls exhibit 3.5 ± 0.3 projections and *dfmr1* mutants 9.2 ± 0.9 (*p* = 2.6 × 10^−6^, two-tailed unpaired *t* test; Fig. 3b, right). The synaptic overgrowth in FXS models may occur during initial synapse formation, or later as a failure to properly prune synapses [44–47]. Our results indicate GFI overgrowth begins at early synaptogenesis stages, but does not rule out a role for faulty pruning later. Some FXS reports show that early synaptic overgrowth is rectified in adults [47–49], so we next assayed whether the excess GFI projections persist in the mature circuit.

### Overgrown axonal projections contain chemical synapse machinery

**Fig. 4:**
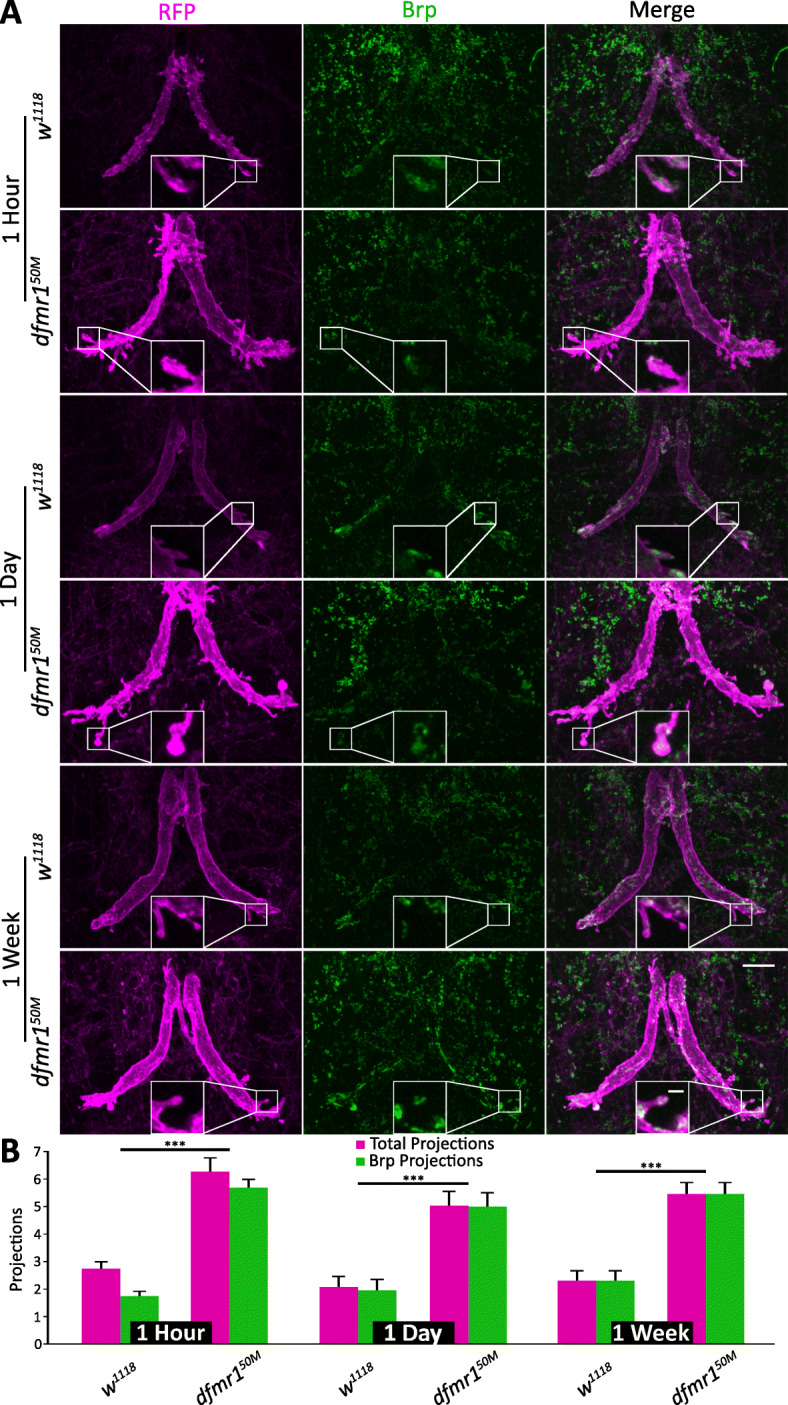
The GFI axonal projections contain chemical synapse markers. **a** The giant fiber interneuron (GFI) axonal bend co-labeled with 91H05-Gal4 driven mCD8::RFP (magenta, column 1) and STaR transgenic labeling of Bruchpilot (Brp) in presynaptic active zones (green, column 2). The merge reveals axonal projections with chemical synapses (column 3). Background controls (*w*^*1118*^, top) and *dfmr1* null mutants (*dfmr1*^*50M*^, bottom) assayed at 1 h, 1 day, and 1 week post-eclosion. Insets show magnified Brp-positive axon projections. Scale bars, 10 μm (full image) and 2 μm (inset). **b** Quantification of total (magenta) and Brp-positive (green) projections for all 3 time points. Significance bars represent comparisons between each genotype for the two projection quantifications for each time point. Sample sizes: 1 h: *w*^*1118*^, *n* = 17; *dfmr1*^*50M*^, *n* = 12; 1 day: *w*^*1118*^, *n* = 12; *dfmr1*^*50M*^, *n* = 13; 1 week: *w*^*1118*^, *n* = 8; *dfmr1*^*50M*^, *n* = 8

Synaptic markers are notoriously difficult to image in dense connectivity regions such as the GFI thoracic ganglia neuropil [50, 51]. Antibody labeling for synaptic markers paired with standard confocal microscopy does not provide sufficient spatial resolution to distinguish whether the synaptic marker is in a neuron of interest or neighboring neurons [52, 53]. Another commonly used approach, the Gal4/UAS transgenic expression of labeled presynaptic markers in neurons of interest, often leads to overexpression which can cause mis-localization and protein aggregation [50, 51, 54]. To avoid these imaging difficulties, we employ here the newer synaptic tagging with recombination (STaR) technique to label Bruchpilot (Brp), a well-studied presynaptic active zone scaffold organizer [50, 55]. STaR labeling requires a stop codon flanked by Flp recombination target (FRT) sites followed by a GFP sequence that is inserted downstream of a protein of interest (Brp-FSF-GFP; Fig. 4). Separately, the flippase (UAS-*flp*) is expressed in the neuron of interest to remove the FRT sites and enclosed stop codon, thus permitting readthrough from Brp to GFP (Brp::GFP; Fig. 4). We use this technique to label presynaptic active zones specifically within the GFI to examine the axonal bend projections.

We took advantage of this STaR labeling method to determine if the presynaptic Brp active zone scaffold is present in the GFI axonal bend projections and to assay the maintenance of these synaptic projections from eclosion through adult maturity (Fig. 4). For these analyses, 91H05-Gal4 was used to drive expression of both membrane mCD8::RFP and Flp to create the GFP-labeled Brp in the marked GFI (Fig. 4a). The background control *w*^*1118*^ and *dfmr1*^*50M*^ null mutant animals were assayed immediately post-eclosion (1 h), during an early activity-dependent refinement period (1 day), and at full adult maturity (1 week), to assay for the persistence of GFI synaptic projections throughout life (Fig. 4a [32]). Results show that the majority of background control and *dfmr1* mutant projections contain the Brp presynaptic scaffold, indicating chemical synapse active zones in presynaptic processes (Fig. 4a). The small Brp::GFP puncta (green) are clearly visible in GFI projections (magenta), both along the projection shafts and at the projection tips (Fig. 4a, inset). Note that while Brp labeling is strong in the GFI, there is signal in surrounding cells. This is likely due to the STaR technique revealing neurons with weak/transient Flp expression. The exogenous signal could come from cells with very low Gal4 activity or with previous expression, including precursor cells. The total synaptic projection number appears to remain steady from 1 h post-eclosion to maturity at 1 week after eclosion (Fig. 4a), suggesting either that these projections are being created and removed at the same rate or, more likely, that projections make stable mature synapses that persist throughout adulthood [56].

Quantification of the synaptic projections shows that 1-h animals have both Brp-negative and Brp-positive processes, albeit with the majority containing chemical synapses (Fig. 4b). In both of these categories, *w*^*1118*^ controls have far fewer projections compared to *dfmr1* nulls (*w*^*1118*^: total projections/bend 2.7 ± 0.3, Brp + projections/bend 1.7 ± 0.2; *dfmr1*^*50M*^: total projections/bend 6.3 ± 0.5, *p* = 1.4 × 10^−5^, two-tailed unpaired *t* test; Brp + projections/bend 5.8 ± 0.3, *p* = 2.7 × 10^−9^, two-tailed unpaired *t* test, Fig. 4b). By 1 day, nearly all projections were Brp positive in both genotypes, with far more synaptic projections in the mutants (*w*^*1118*^: total projections/bend 2.1 ± 0.4, Brp + projections/bend 2.0 ± 0.4; *dfmr1*^*50M*^: total projections/bend 5.1 ± 0.6, *p* = 2.0 × 10^−4^, two-tailed unpaired *t* test, Brp + projections/bend: 5.0 ± 0.5, *p* = 2.8 × 10^−6^, two-tailed unpaired *t* test, Fig. 4b). At 1 week, projection numbers were similar to 1 day, and every projection has chemical synapses in both genotypes, with more in the mutants (*w*^*1118*^: Brp + projections/bend 2.3 ± 0.4; *dfmr1*^*50M*^: Brp + projections/bend 5.5 ± 0.5, *p* = 1.1 × 10^−4^, two-tailed unpaired *t* test; Fig. 4b). Together, these findings suggest axonal projections that extend from the GFI bend make chemical synaptic connections with postsynaptic partners. As the GFI uses mixed chemical and electrical synapses, we next tested whether the supernumerary axonal projections also contain electrical synapses [57].

### Overgrown axonal projections contain electrical synapses

**Fig. 5:**
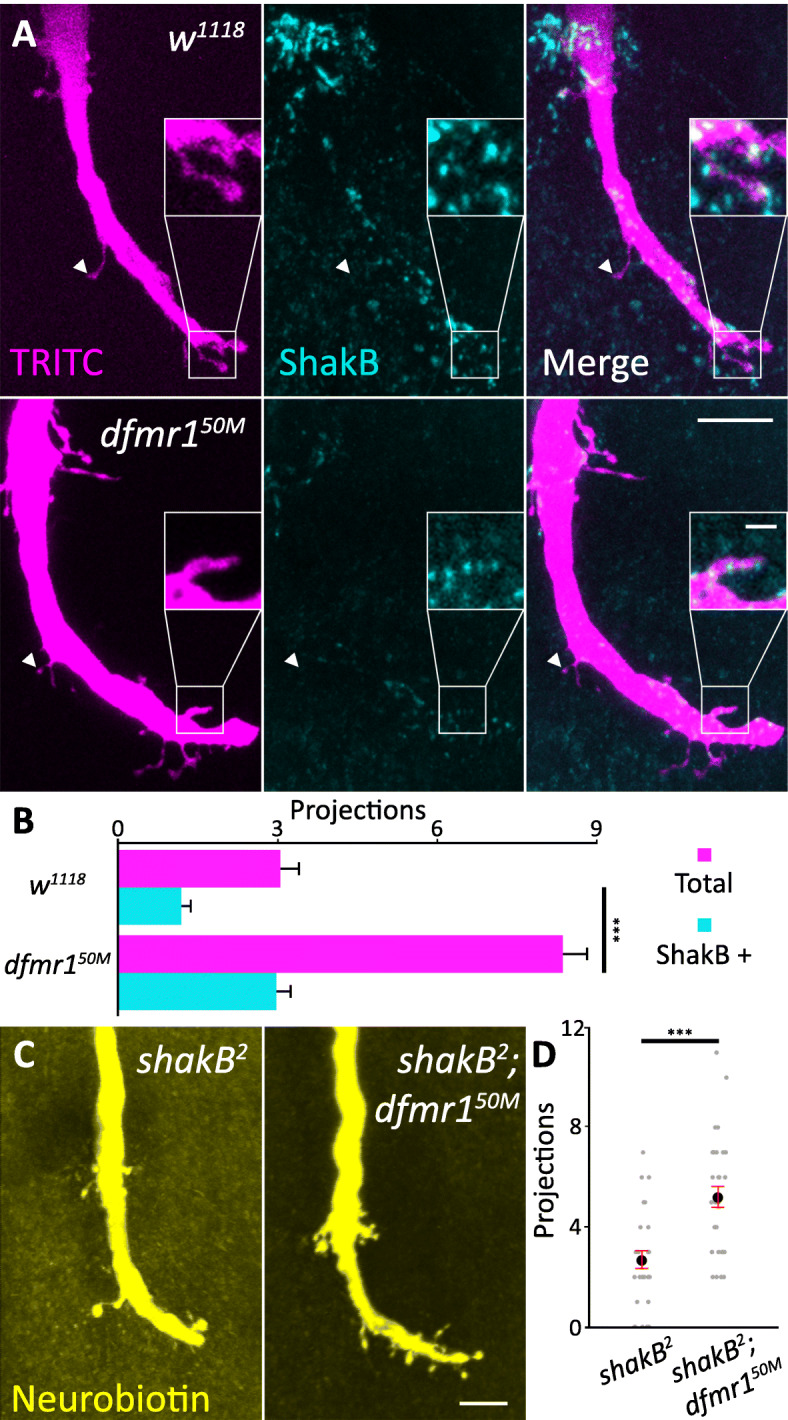
The GFI axonal projections contain electrical synapse markers. **a** Giant fiber interneuron (GFI) TRITC-dextran dye injected (magenta, left) and labeled for ShakB (cyan, center) reveals axonal bend projections containing electrical synapses (merge, right) in w^1118^ (top) and dfmr1^50M^ (bottom). Arrowheads indicate ShakB-negative projections; insets show magnified ShakB-positive projections. Scale bars, 10 μm (full image) and 2 μm (inset). **b** Quantification of total (magenta) and ShakB-positive (cyan) projections for both genotypes. Significance bars represent comparisons between each genotype for the two projection quantifications. Sample sizes: w^1118^, *n* = 28; *dfmr1*^*50M*^, *n* = 26. **c** GFI dye-injected with Neurobiotin (yellow) in shakB^2^ null mutant alone (left) and the shakB^2^; dfmr1^50M^ double mutant (right). Scale bar, 10 μm. **d** Quantification of projections for both genotypes. Each gray dot represents the projection number for an axon bend in one animal. The black dot represents the mean and red bars represent the standard error of the mean. Sample sizes: s*hakB*^*2*^, *n* = 27; *shakB*^*2*^*; dfmr1*^*50M*^, *n* = 31

Unlike general synaptic antibodies, the Shaking-B (ShakB) antibody specifically labels GFI electrical synapses, permitting simple imaging analyses [58]. To test whether GFI axon projections electrically couple to partner neurons, the GFI axon was injected with TRITC-dextran and labeled with the ShakB antibody (Fig. 5a). Unlike Brp, we find ShakB present in a limited subset of projections (Fig. 5a, insets), with many projections either negative or below detection limits (Fig. 5a, arrowheads). Quantification shows that both *w*^*1118*^ controls and *dfmr1* nulls have ShakB in less than half of the GFI synaptic projections (Fig. 5b). The *dfmr1*^*50M*^ animals exhibit projection overgrowth, both for total and ShakB+ projections (*w*^*1118*^: total projections/bend 3.0 ± 0.4, ShakB+ projections/bend 1.2 ± 0.2; *dfmr1*^*50M*^: total projections/bend 8.3 ± 0.5, *p* = 2.1 × 10^−11^, two-tailed unpaired *t* test, ShakB+ projections/bend 3.0 ± 0.3, *p* = 6.1 × 10^−6^, two-tailed unpaired *t* test; Fig. 5b). We found no significant difference in ShakB signal levels or punctae number between *w*^*1118*^ and *dfmr1*^*50M*^, as previously reported [59]. These results suggest that while axonal projections clearly can form electrical synapses, this is not a universal mode of connectivity. We next wanted to test whether GFI projections depend on electrical synapses for formation or maintenance, as many vertebrate synapses have been reported to use gap junctions during early synaptogenesis, which are then removed when synapse formation is complete [60, 61].

The GFI electrical synapses can be eliminated using the s*hakB*^*2*^ null mutant [57]. We tested this mutant alone and combined with *dfmr1*^*50M*^, injecting the GFI with gap junction permeable NB (Fig. 5c). Dye injections were performed for 30 s, as longer injections cause s*hakB*^*2*^ neurons to rupture [59]. In these experiments, no neurons aside from the GFI are labeled, indicating the successful removal of ShakB. Both single and double mutants still produce projections (Fig. 5c, left), though *shakB*^*2*^*; dfmr1*^*50M*^ maintains overgrown projections relative to control (Fig. 5c, right). Quantification shows that *dfmr1*^*50M*^ has a significant increase in projections (projections/bend: *shakB*^*2*^ − 2.7 ± 0.4; *shakB*^*2*^*; dfmr1*^*50M*^ − 5.2 ± 0.4, *p* = 5.2 × 10^−5^, two-tailed unpaired *t* test; Fig. 5d). These results suggest that some *dfmr1*^*50M*^ projections could be ShakB electrical synapse dependent, but certainly not all of them. Taken together, the above findings show that the GFI axonal projections contain both chemical (Brp) and electrical (ShakB) synaptic markers. The extent that these synaptic projections travel away from the GFI axon bend suggests they connect with postsynaptic partners other than the TTMn, which lays tightly along the GFI axon [57]. We therefore next identified the mutant synaptic partners to determine if they are known GF circuit neurons or new, inappropriate targets.

### Synaptic projections target GFC neurons to cause GF circuit hyperconnectivity

**Fig. 6:**
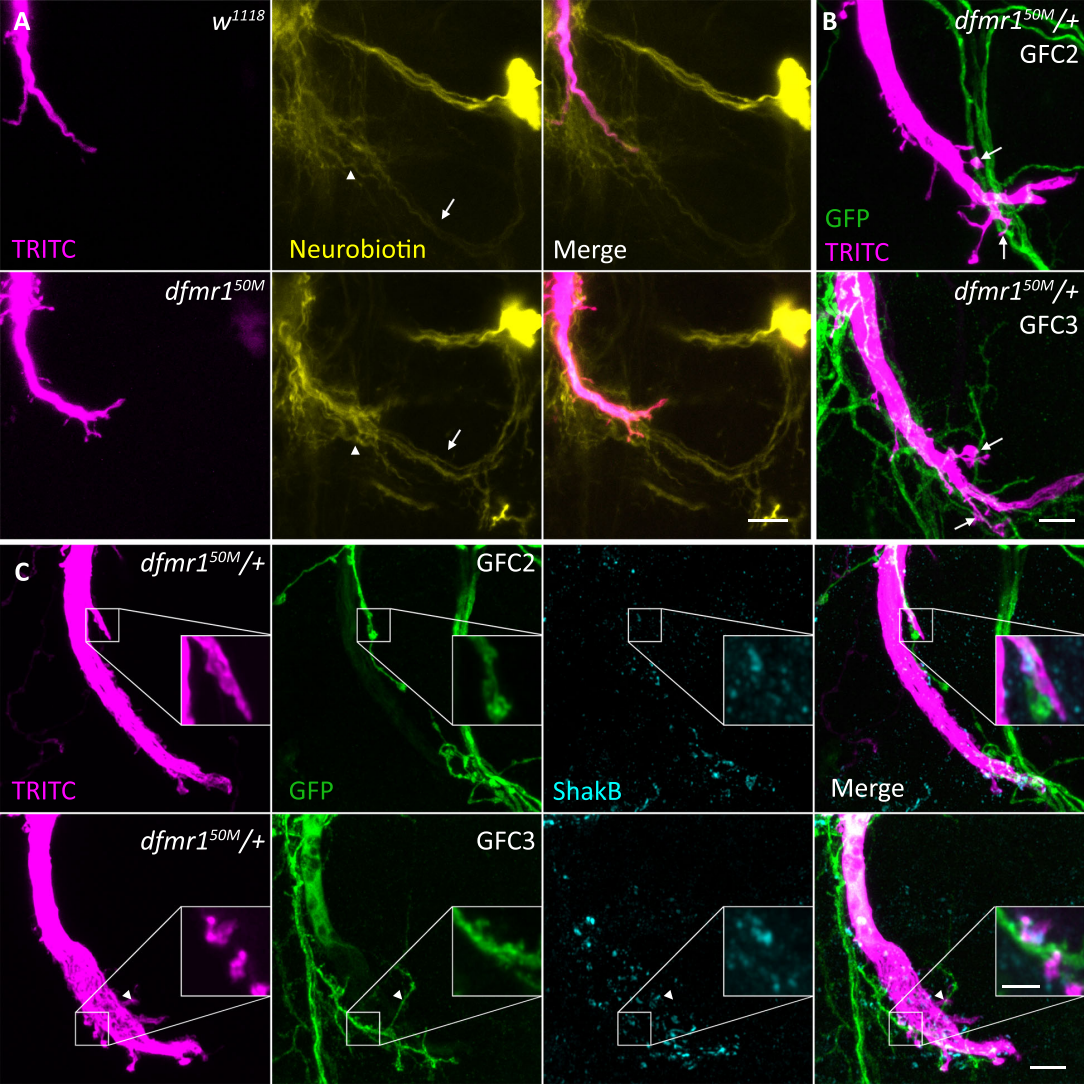
GFI synaptic projections connect within the GF circuit on GFC neurons. **a** Giant fiber interneuron (GFI) dye co-injected with TRITC-dextran (magenta, left) and neurobiotin (yellow, center) to assay downstream neurons (merge, right) in *w*^*1118*^ (top) and *dfmr1*^*50M*^ (bottom). Presumed GFC2 (arrow) and GFC3 (arrowhead) are contacted by GFI projections. Scale bar, 10 μm. **b** GFI injected with TRITC-dextran (magenta) with 73C07-Gal4 driving mCD8::GFP (green) in GFC2 (top), and 24H07-Gal4 driving mCD8::GFP (green) in GFC3 (bottom) in the *dfmr1*^*50M*^/+ background. Arrows point to overlaps between GFI and GFCs. Scale bar, 5 μm. **c** GFI injected with TRITC-dextran (magenta, column 1) with mCD8::GFP (green, column 2) labeling GFC2 (73C07-Gal4, top) and GFC3 (24H07-Gal4, bottom) co-labeled for the ShakB innexin (cyan, column 3) to reveal electrical synapses in the *dfmr1*^*50M*^/+ background. All three channels are combined in the merge (column 4). Insets show magnified sub-stacks of ShakB-positive GFI synaptic projections contacting GFC neurons. The arrowhead shows an example ShakB-negative GFI-GFC contact. Scale bars, 5 μm (full image) and 2 μm (inset). All images taken in Airyscan mode for increased resolution

**Fig. 7:**
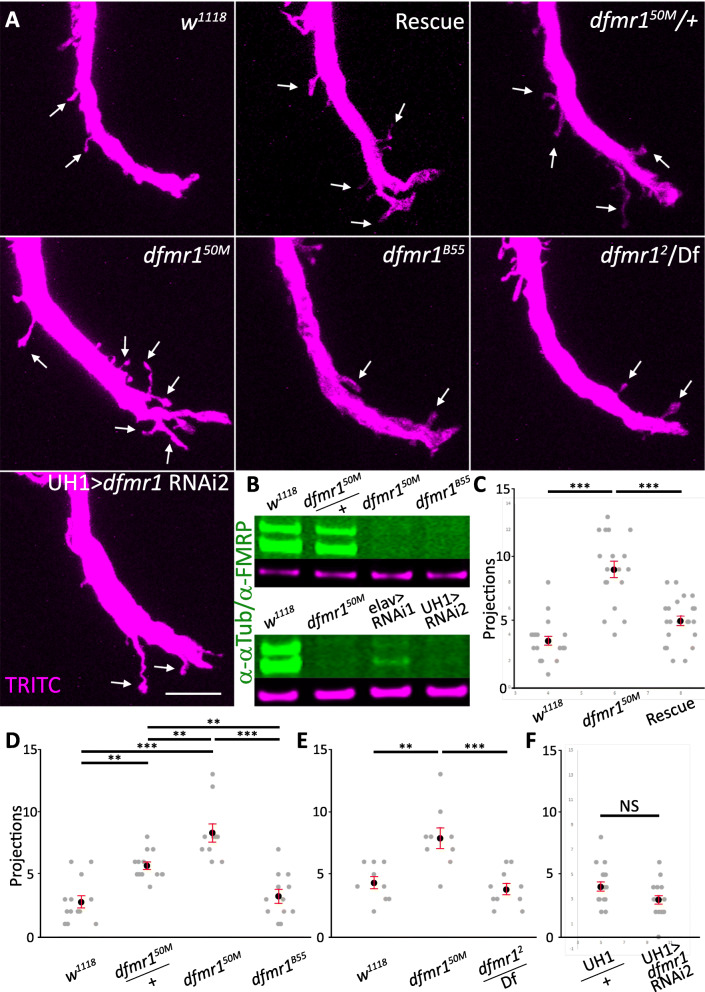
GFI synaptic projections dependent on *dfmr1*^*50M*^ background mutations. **a** Giant fiber interneuron (GFI) TRITC-dextran dye injected (magenta) in the indicated genotypes; genetic background control (*w*^*1118*^), genomic rescue (*dfmr1.14*/+*; dfmr1*^*50M*^), *dfmr1*^*50M*^ heterozygote (*dfmr1*^*50M*^/+), homozygous null mutant *(dfmr1*^*50M*^), independent *dfmr1* null (*dfmr1*^*B55*^), and second independent *dfmr1* null over a deficiency (*dfmr1*^*2*^/Df). FMRP was also removed using RNAi driven by the ubiquitous *daughterless* Gal4 driver (UH1; UH1>*dfmr1* RNAi2). Arrows indicate projections. Scale bar, 10 μm. **b** Western blot of FMRP levels in *w*^*1118*^, *dfmr1*^*50M*^/+, *dfmr1*^*50M*^, and *dfmr1*^*B55*^ (top); and *w*^*1118*^, *dfmr1*^*50M*^, *elav*>*dfmr1* RNAi1 and UH1>*dfmr1* RNAi2 (bottom). FMRP bands are labeled in green and α-Tubulin loading controls in magenta. **c** Quantification of projections in *w*^*1118*^ (*n* = 20), *dfmr1*^*50M*^ (*n* = 18), and the *dfmr1* rescue condition (*n* = 22). **d** Quantification of projections in *w*^*1118*^ (*n* = 13), *dfmr1*^*50M*^/+ (*n* = 13), *dfmr1*^*50M*^ (*n* = 10), and *dfmr1*^*B55*^ (*n* = 12). **e** Quantification of projections in *w*^*1118*^ (*n* = 9), *dfmr1*^*50M*^ (*n* = 9), and *dfmr1*^*2*^/Df (*n* = 10). **f** Quantification of projections in UH1/+ control (*n* = 17) and UH1>*dfmr1* RNAi2 (*n* = 16). Each gray dot represents the projection number for an axon bend in one animal. The black dot represents the mean and red bars represent the standard error of the mean

The GF circuit connectivity can be mapped by injecting small tracers that pass through gap junctions to label the partner neurons [36]. We took advantage of this property by injecting the GFI axon with NB to test whether new, out-of-circuit neurons partner with supernumerary *dfmr1*^*50M*^ synaptic projections [29, 37]. The *w*^*1118*^ control and *dfmr1*^*50M*^ null dye coupling patterns, although complex, are extremely similar, with no newly labeled neurons appearing in the mutant condition (Fig. 6a). Moreover, upon close analyses of the *dfmr1*^*50M*^ projection locations, it appears that they contact recently identified neurons within the GF circuit, specifically GFC2 (Fig. 6a, arrow) and GFC3 (Fig. 6a, arrowhead [29]). Since transgenic tools are available to study these neuron classes, we tested whether the mutant projections are overgrown on these normal GFI targets. We combined the GFC2 (73C07-Gal4) and GFC3 (24H07-Gal4) drivers with UAS-*mcd8::gfp* and crossed these animals with the *dfmr1*^*50M*^ stock, since *dfmr1*^*50M*^/+ increases projection number (see Fig. 7 below). We injected the GFI with TRITC to find that GFI projections frequently oppose both GFC2 and GFC3 neurons (Fig. 6b, arrows), indicating putative synaptic connectivity. We next wished to test whether these direct contacts are incidental or indicate synaptic pairing.

To test synaptic connectivity, we repeated the above dye injection experiments while co-labeling for the ShakB innexin to identify electrical synapses (Fig. 6c). We find that ShakB synapses are present at the contact intersection of the GFI projections and the GFC2/3 neurons (Fig. 6c, insets), although there are also frequently cases where ShakB labeling is not detectable in the GFI synaptic projections contacting a GFC neuron (Fig. 6c, arrowhead). Taken together, these findings suggest that the synaptic projections characterizing the *dfmr1*^*50M*^ mutant GFI make redundant connections onto other known GF circuit neurons. However, we cannot definitively state that GFC2 and 3 are the only synaptic targets of the excess projections. Moreover, it is interesting to note that GFC2 and 3 extensively contact the main axon shaft in both controls and *dfmr1* nulls and form synapses there [29], making the projections unnecessary. This finding suggests either that the mutant condition drives the axon to seek out more synapses with its partners than it normally would require or that developmental projections normally pruned away during GF circuit maturation are inappropriately stabilized (Fig. 3). In pursuit of this question, we uncovered evidence that suggested FMRP loss may not cause the synaptic projection phenotype, so we pursued a series of control experiments to test genetic background effects.

### FXS disease model hyperconnectivity depends on background mutations

To ensure that FMRP loss was responsible for the excess GFI axonal synaptic projections, we performed transgenic rescue experiments and tested alternative *dfmr1* null mutants for the GFI phenotype. For the rescue experiment, we used the full length genomic *dfmr1* sequence, including the full regulatory region, inserted onto the second chromosome (*dfmr1.14* [62]). For the alternative nulls, we examined the homozygous viable *dfmr1*^*B55*^ allele, as well as the *dfmr1*^*2*^ allele over a deficiency (Df (3R)BSC621), which completely removes *dfmr1* and numerous adjacent genes [62–64]. Both *dfmr1*^*B55*^ and *dfmr1*^*2*^ are reported to be complete protein nulls in the adult brain, although *dfmr1*^*B55*^ has been found to express FMRP in the testes [18]. We also tested the heterozygous *dfmr1*^*50M*^ condition (*dfmr1*^*50M*^/+) to determine whether full protein loss is required for the phenotype or if the defect occurs in heterozygotes, as has been reported previously in the *Drosophila* FXS model [65]. Finally, to further test FMRP loss effects on synaptic projection overgrowth we took a transgenic RNAi approach using a highly expressing ubiquitous Gal4 driver to express a characterized *dfmr1* RNAi (UH1-Gal4>*dfmr1* RNAi2 [66]). All of these studies are summarized in Fig. 7.

We first tested the genetic rescue condition (*dfmr1.14*/+; *dfmr1*^*50M*/*50M*^ = “rescue”) by injecting the GFI with TRITC-dextran to assay the synaptic projection number. The re-introduction of wildtype FMRP causes a partial correction of the *dfmr1*^*50M*^ phenotype, with significantly fewer synaptic projections present compared to the null mutant (projections/bend: *dfmr1*^*50M*^ − 9.0 ± 0.6, rescue − 5.1 ± 0.4; unpaired ANOVA with Tukey post hoc analysis, *p* = 2.7 × 10^−7^; Fig. 7a, c). This single *dfmr1* copy rescue is not complete, as more synaptic projections occur in the rescue condition than in the *w*^*1118*^ genetic background control (3.6 ± 0.3 projections/bend), and this difference is significant (unpaired ANOVA with Tukey post hoc analysis, *p* = 0.047; Fig. 7a, c). Importantly, similar to the wildtype FMRP rescue results, single copy *dfmr1*^*50M*^/+ heterozygotes also show an intermediate excess synaptic projection defect (projections/bend: *w*^*1118*^ − 2.8 ± 0.5, *dfmr1*^*50M*^/+ − 5.7 ± 0.5, *dfmr1*^*50M*^ − 8.3 ± 0.7), with significant differences in all the comparisons (unpaired ANOVA with Tukey post hoc analysis: *w*^*1118*^*v. dfmr1*^*50M*^/+ *p* = 0.001, *dfmr1*^*50M*^/+ vs. *dfmr1*^*50M*^*p* = 0.006; *w*^*1118*^ vs. *dfmr1*^*50M*^*p* = 2.5 × 10^−8^; Fig. 7a, d). Taken together, these results support the conclusion that FMRP loss causes the excess production of supernumerary GFI synaptic projections.

Surprisingly, however, the alternate *dfmr1* mutants did not replicate the synaptic projection phenotype. The homozygous *dfmr1*^*B55*^ mutants appear nearly identical to control animals (projections/bend: *w*^*1118*^ − 2.8 ± 0.5, *dfmr1*^*B55*^ − 3.3 ± 0.6), with no significant difference seen (unpaired ANOVA with Tukey post hoc analysis, *p* = 0.9; Fig. 7a, d). We tested *dfmr1*^*B55*^ by Western blot and confirmed that no FMRP is detectably expressed in the brain (Fig. 7b). The *dfmr1*^*2*^/Df test, carried out separately, shows the same result, with no GFI synaptic projection increase relative to controls (projections/bend: *w*^*1118*^ − 4.3 ± 0.5, *dfmr1*^*2*^/Df − 3.8 ± 0.5, *dfmr1*^*50M*^ − 7.9 ± 0.8, unpaired ANOVA with Tukey post hoc analysis, *p* = 0.8; Fig. 7a, e). Finally, ubiquitous RNAi FMRP knockdown also does not increase GFI synaptic projections compared to the transgenic control (projections/bend: UH1-Gal4/+ control − 4.1 ± 0.4, UH1-Gal4>*dfmr1* RNAi2 − 3.0 ± 0.4), with no significant difference in projection number (two tailed unpaired *t* test, *p* = 0.05; Fig. 7a, f). Western blot analyses show that ubiquitous UH1-Gal4 driven *dfmr1* RNAi2 completely eliminates detectable FMRP from the brain, in contrast to an alternate knockdown approach of pan-neuronal *elav*-Gal4 driven *dfmr1* RNAi1, which shows the maintenance of a weak, residual FMRP signal in the brain (Fig. 7b).

To further test the above apparent semi-dominance observed with *dfmr1*^*50M*^/+ heterozygotes, we carried out additional analyses with the well-characterized *dfmr1*^*3*^ null allele [62]. A second transheterozygous *dfmr1* test was done by pairing the *dfmr1*^*50M*^ allele with the *dfmr1*^*3*^ allele. The results show control projections (2.8 ± 0.4/bend, *n* = 18) are significantly lower than *dfmr1*^*50M*^/+ heterozygotes (5.9 ± 0.4/bend, *n* = 17, unpaired ANOVA with Tukey post hoc analysis, *p* = 3.8 × 10^−5^), and also significantly reduced compared to the *dfmr1*^*50M*^/*dfmr1*^*3*^ trans-heterozygotes (6.8 ± 0.5/bend, *n* = 16, unpaired ANOVA with Tukey post hoc analysis, *p* = 1.8 × 10^−7^). Trans-heterozygotes were not significantly different than heterozygotes (ANOVA with Tukey post hoc analysis, *p* = 0.47. All three of these genotypes are significantly different compared to the homozygous *dfmr1*^*50M*^*/dfmr1*^*50M*^ mutant condition (8.7 ± 0.5 projections/bend, *n* = 15, unpaired ANOVA with Tukey post hoc analysis, *p* = 2.1 × 10^−11^, 2.5 × 10^−4^, and 0.02, respectively). We note that there is overlap in the *w*^*1118*^ control, *dfmr1*^*50M*^/+ heterozygote and *dfmr1*^*50M*^*/dfmr1*^*50M*^ homozygous animals used in the quantification of this experiment and the above *dfmr1*^*B55*^ experiment due to a shift in experimental design resulting from poor availability of weakened animals. Nevertheless, the additional results with the independent *dfmr1*^*3*^ null allele serve to confirm and extend the conclusions from the above *dfmr1*^*50M*^, *dfmr1*^*B55*^ and *dfmr1*^*2*^ studies.

Taking all of the above results together, we conclude that FMRP loss by itself does not cause the excess synaptic projections from the GFI axonal bend and that a second site mutation(s) in the *dfmr1*^*50M*^ genetic background is required for the GF circuit hyperconnectivity defect. Importantly, the wildtype FMRP rescue was misleading in this case, causing us to draw the incorrect initial conclusion of a sole FMRP-specific requirement. The simple interpretation was that the rescue reinserted a single wildtype *dfmr1* allele into a null background to provide partial phenotype restoration. However, since alternative *dfmr1* null mutant and *dfmr1* RNAi lines lack the synaptic projection phenotype, it may be that a background mutation was lost when *dfmr1.14* was combined with *dfmr1*^*50M*^. We do not rule out a role for FMRP, as it could be acting in concert with the background mutation(s) to enhance the synaptic projection phenotype. Interestingly, FXS patients also show similar genetic background effects, as the severity of the disease symptoms present over a very wide spectrum [23–25]. We therefore chose to pursue the FXS model background mutation(s) as a way to shed new light on molecular players in synapse development that could include novel FMRP interactors.

### Identifying FXS background mutations driving intra-circuit hyperconnectivity

**Fig. 8:**
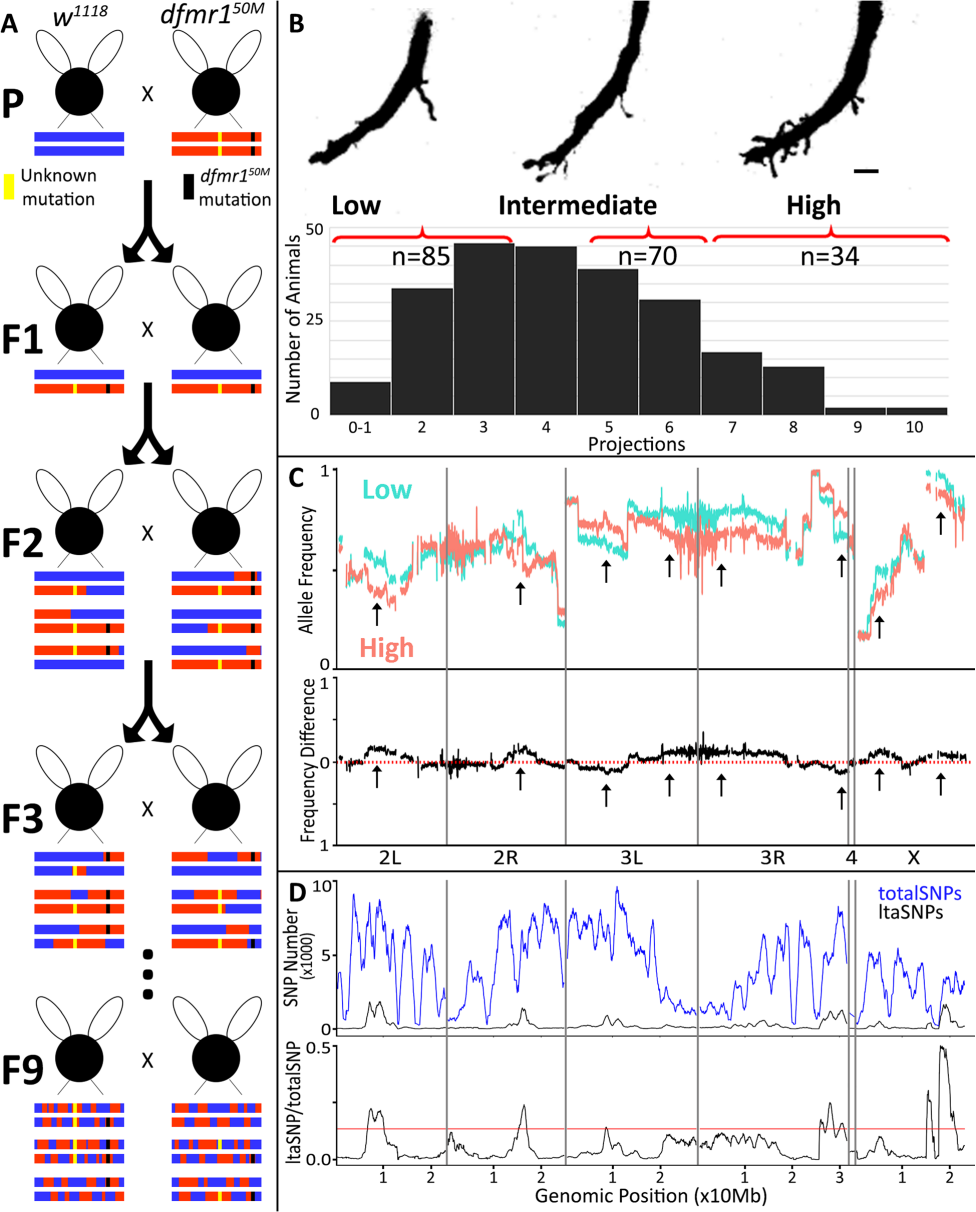
Bulked segregant analysis of synaptic projection quantitative trait loci. **a** BSA performed by crossing mutant (*dfmr1*^*50M*^) with the paired control (*w*^*1118*^) to create a heterozygous F1 generation. Successive interbreeding of offspring for ≥ 9 generations generated recombinant inbred lines. Schematic recombinants are represented for *w*^*1118*^ background (blue), mutant background (red), *dfmr1*^*50M*^ deletion (black), and background mutation (yellow). **b** Top: The recombinant offspring segregated into three pools of low (0–3 projections), intermediate (5–6), and high (7+) GFI projection phenotype classes. Scale bar represents 5 μm. Bottom: The full distribution of GFI projection numbers from 234 GFI single-cell injections, pooled into phenotype classes. **c** Top: Low (light blue) and high (light red) phenotype classes plotted for *w*^*1118*^ SNP frequency, averaged in 100 kb windows across the four *Drosophila* chromosomes: 2 L/R, 3 L/R, 4, and X. Bottom: Difference in *w*^*1118*^ SNP frequency between the low and high phenotype classes. Arrows represent regions of divergence. **d** Top: The total combined SNPs (totalSNPs) for both bulks (blue) and the likely trait-associated SNPS (ltaSNPs, black) for both bulks plotted in 2 Mb sliding windows with 10 kb steps. Bottom: Ratio of ltaSNPs to totalSNPs, with a red line indicating the 99% confidence interval

We employed bulked segregant analysis (BSA) paired with whole genome sequencing (WGS) to identify *dfmr1*^*50M*^ background mutations. BSA has been used to identify de novo mutations from divergent backgrounds in many systems, including *Drosophila* [35, 67–70]. We pursued BSA by first inbreeding the heterozygous offspring of a *w*^*1118*^ X *dfmr1*^*50M*^ cross for 9 generations (Fig. 8a). We then analyzed GFI synaptic projection number by intracellular dye injection, sorting animals into low, medium, and high phenotype pools (Fig. 8b). Inbreeding was continued during analysis with animals selected from the 9th–12th generations. A total of 234 animals were analyzed by single-neuron dye injection, from which 85 were in the low projection pool (0–3 projections), 70 in the medium pool (5–6 projections), and 34 in the high pool (7+ projections, Fig. 8c). An additional 45 animals had 4 projections, but these were not included in the analysis to keep the low and medium pools distinct. The intermediate pool was designed to capture partial phenotypes that might arise from intermixing of *w*^*1118*^ and *dfmr1*^*50M*^. The DNA extracted for each pool was used for WGS analysis to identify enriched genomic regions in the high pool relative to the low pool. The average BSA sequencing depths were 146 for the low pool and 112 for the high pool. The parental lines were sequenced separately and had average depths of 18 for *w*^*1118*^ and 11 for *dfmr1*^*50M*^ [71].

We analyzed bulked segregation to assay for significant changes ([72], J. Wang, personal communication). We mapped frequencies of control *w*^*1118*^ SNPs at each variable position in the low and high bulks (Fig. 8c, top). If a SNP is not linked, we expected equal representation of *w*^*1118*^ and *dfmr1*^*50M*^ parental reads. In contrast, causative SNPs should be enriched in the high bulk and lost in the low bulk due to phenotype selection. When SNP frequencies are mapped, two outcomes appear. First, several divergences occur between low and high pools, suggesting regions linked to the phenotype. These occur on all chromosomes, except chromosome 4 (Fig. 8c, top, arrows). Divergences are more apparent when the frequencies for the high bulk are subtracted from the low bulk, and the difference plotted (Fig. 8c, bottom, arrows). Second, low and high pools often veer sharply away from the predicted 50/50 distribution between *dfmr1*^*50M*^ and *w*^*1118*^, typically favoring *w*^*1118*^. This is obvious towards the end of chromosome 3R, where both bulks approach *w*^*1118*^. We expect atypical distributions are due to selection against mutations that hinder viability or fecundity. Indeed, *dfmr1*^*50M*^ animals are sterile with reduced viability; traits attributed to FMRP loss, but which could also depend on background effects [21, 62, 73, 74]. Interestingly, other regions have *w*^*1118*^ sequence disfavored, such as the start of the X chromosome.

A variety of statistical methods have been developed for analyzing BSA data [75–77]. We first used the QTLseqr method to define quantitative trait loci (QTL), which applies two separate statistical tests (QTL-seq and G’ [76, 78, 79]). Both statistical tests show that the divergent peaks present in the allele frequency analyses are enriched in the high phenotype pool, although they do not reach significance (data not shown). To further test the BSA data, we next analyzed results using MULTIPOOL, a different platform that uses Bayesian-based statistical inference [75]. This method also identified the same divergent regions as individual peaks, but similar to the QTLseqr analysis, found the segments are not significantly different (data not shown). As a third test, we applied the most recently developed PyBSASeq platform to our BSA dataset analysis [77]. This method uniquely tests for SNP enrichment across all chromosomal intervals, rather than at individual sites, to increase the QTL detection sensitivity [77]. This improved PyBSASeq analysis identified multiple statistically significant QTLs linked to the intra-circuit synapse hyperconnectivity phenotype. Comparisons were done only between the high and low phenotype pools, as including the intermediate pool data did not help refine the comparison further. Importantly, the same QTLs were identified with all three analysis methods.

The first step in PyBSASeq processing is to identify individual SNPs where the low and high bulks are significantly different (*p* < 0.001) based on a Fisher’s exact test. These “likely trait associated SNPS” (ltaSNPs) are plotted in sliding windows of 2 Mb with 10 kb steps. When total SNPs (blue) and ltaSNPs (black) are plotted for BSA data, peaks emerge that correspond with the previously seen allele frequency divergences (Fig. 8d, top). To normalize for SNP density, the ltaSNPs to total SNPs ratio is plotted (Fig. 8d, bottom), showing strong peaks past the 99% confidence interval (red line), indicating causative QTLs. The identified peaks are in the following regions: 2 L:6.72–10.19; 2R:15.27–16.98; 3 L:8.56–8.91; 3R:25.79–26.7, 27.2–28.84 and 30.0–30.99; X:15.14–16.27, 17.68–21.17 (all values in Mb). All genes and miRNAs within these loci are listed in Additional File 2. The smallest of these ranges, 3 L:8.56–8.91, includes 351 ltaSNPs in a genomic region that contains 45 protein-coding genes and 1 miRNA, demonstrating the resolution is not sharp enough to identify causative mutations from these results. Nevertheless, these 8 QTLs represent discrete regions of the genome related specifically to the synaptic hyperconnectivity phenotype identified in this study. The regions are small enough that they can be probed in the future with linkage analysis, complementation assays, and/or candidate RNAi screens [80–82].

## Discussion

The goal of this work was to exploit the particularly well-mapped giant fiber circuit in order to dissect mechanisms underlying the FXS hallmark phenotype: supernumerary synapses [15, 21, 48, 83, 84]. While striking synaptic overgrowth did indeed manifest in this model circuit, causing intra-circuit hyperconnectivity, the defect is driven by background mutations in a *dfmr1*^*50M*^ stock. FMRP loss was ruled out as the cause of GFI synaptic overgrowth based on three separate genetic elimination strategies: (1) *dfmr1* RNAi, (2) independent *dfmr1* alleles, and (3) genomic deficiency trans-heterozygotes [32, 62–64]. However, the supernumerary synapse phenotype was significantly rescued by the re-introduction of wildtype FMRP [62], suggesting an FMRP requirement. Based solely on rescue results, it would appear that FMRP loss partially contributes to circuit hyperconnectivity, interacting with genetic background mutations. Indeed, unidentified background mutations are widely reported to interact with FMRP loss in the mouse FXS model, creating learning defects and exacerbating autism-like behaviors [85–87]. Similarly, FXS patients show a broad disease symptom severity spectrum dependent on genetic background modifiers [86, 88].

However, in light of the independent *dfmr1* null mutant results, FMRP rescue of the intra-circuit hyperconnectivity phenotype requires close scrutiny. Genetic rescue is interpreted as the strongest evidence of a gene-specific requirement, but background mutations can complicate this interpretation: recombining *dfmr1*^*50M*^ and rescue lines may outcross contributing mutations. Indeed, recombined *dfmr1*^*50M*^ animals always have fewer GF synaptic projections than the original stock (Figs. 2a, 4a, 5c vs. Figs. 2b, 5a, 6a). It should also be noted that *dfmr1*^*50M*^ was not enriched in the bulked segregant analysis, suggesting it is not required for the synaptic phenotype. Thus, the FMRP role in intra-circuit hyperconnectivity remains unclear and will require further interaction tests once the background mutations are identified. This work serves as a vital reminder that new *dfmr1* phenotypes should be validated by RNAi or fully independent mutant alleles. In other neural circuits, numerous *dfmr1*^*50M*^ phenotypes have been validated with *dfmr1* RNAi and/or independent *dfmr1* mutants, including the *dfmr1*^*2*^, *dfmr1*^*3*^, and *dfmr1*^*B55*^ alleles used here [18, 89–96], but *dfmr1*^*50M*^ background interactions have also been observed [97]. Studies lacking proper vetting should be validated before becoming the basis of future research. We note that it is unclear when these particular *dfmr1*^*50M*^ background mutations arose, and they may be limited to just this one *dfmr1*^*50M*^ stock. Nevertheless, this work serves as a cautionary tale of the possible effects of genetic background, even in well-controlled studies.

The supernumerary GFI synaptic projections identified here arise from extensive filopodial outgrowths occurring during early synaptogenesis, although reduced synaptic pruning could also play a later contributing role [44, 89, 98]. The projection overgrowths occur throughout the course of giant fiber circuit formation, suggesting there is never a developmental stage where controls and mutant animals have equivalent synaptic outgrowth with subsequent differential pruning. This result supports a model in which increased GFI filopodial outgrowth is later converted to excess mature connections containing both chemical (Brp active zone scaffold) and electrical (ShakB gap junction innexin) synapses [55, 99]. These mixed chemical/electrical synapse connections are a well-characterized feature of the giant fiber escape circuit [57, 100]. Although the great speed benefit of gap junction synapses is obvious for any escape circuit, it is not clear how slower chemical synapses contribute to escape circuit signaling, or indeed are prevented from muddying the fidelity of communication given the differential timing delay. Future studies will also be needed to determine the specific contribution of the axonal synaptic projections to giant fiber neural connectivity.

No new out-of-circuit neurons can be identified as dye-coupled to the mutant GFI due to the excess synaptic projection overgrowths, indicating no inappropriate neural partnerships occur in the mutants. It is possible that inappropriate partners exist in the dense neuropil around GFI axonal bends, with electrical connections too weak to pass sufficient dye for detection [101]. Alternatively, inappropriate partners could be solely linked by chemical synapses. The identified targets of the excess synaptic projections are the GFC2 and GFC3 neurons, newly established giant fiber circuit members [29]. GFC2/3 neurons contact the GFI with en passant synapses at both the more proximal inframedial bridge and more distal axonal bends. GFC2/3 neurons have quite complex architectures, and it is unclear if the GFI synaptic projections contact the same regions in these neurons as en passant connections [29]. Synaptic contact of partner neurons at inappropriate sites, as well as the formation of spatially isolated projection synapses, may deleteriously impact GFI information flow and circuit function [73, 102]. It will be particularly interesting to test how these supernumerary synaptic projections impact giant fiber circuit activity and output escape behavior [103, 104].

Bulked segregant analysis (BSA) paired with whole genome sequencing (WGS) has proven a powerful approach to identify genes in numerous processes [35, 68, 75]. Here, *dfmr1*^*50M*^ and background lines were repeatedly recombined to generate offspring with supernumerary synapses. The phenotype distribution was positively skewed, likely reflecting the nature of the mutations (e.g., dominant vs. recessive), how they interact (additive, synergistic, redundant), and presence of linked deleterious alleles [105–107]. PyBSASeq using pooled single nucleotide polymorphism (SNP) analyses identified eight QTL regions, ranging from 0.35 to 3.49 Mb [77]. QTL identification may have been complicated by a large number of causative mutations. Multiple interacting genetic sites are more difficult to detect using BSA, especially if they act additively or co-dependently [67, 72]. An intermediate bulk pool was included to increase the QTL resolution, but this added no further information, likely due to the complexity of the genetic interactions. Traditional QTL analysis or genome-wide association study (GWAS) of individually sequenced animals with a broad phenotypic range could help isolate the individual mutations and further winnow down the genes in the QTLs identified in this study [108, 109].

Mechanisms driving intra-circuit synaptic hyperconnectivity are suggested by the QTL genes (Additional File 2). As supernumerary projections are apparent early in circuit development, and endure as mature synaptic connections following eclosion, candidate genes are predicted to regulate synapse formation and/or stabilization, rather than activity-dependent refinement [110, 111]. From the gene list (Additional File 2), the gene products most likely to be involved include (1) cytoskeletal regulators responsible for axonal filopodial outgrowth, along with their accessory and regulatory proteins (e.g., Rho, RapGAP [112]), (2) cell adhesion molecules (CAMs) involved in synapse targeting/ initiation (e.g., Liprin-α, Neuroglian [5, 113, 114]), (3) extracellular signaling ligands driving synapse formation and stabilization (e.g., Wingless (Wnt-1) [91, 115, 116]), (4) synaptic destabilization/degradation machinery (e.g., Plum [117]), and (5) regulatory proteins that may control the above mechanisms (e.g., transcription factors, epigenetic modifiers [118, 119]). Although these are the primary candidates of interest, other intriguing genes are present in the 8 QTLs identified here (Additional File 2). Future work using RNAseq or linkage analyses will help identify the genes involved in synaptogenesis and place them in a broader neurodevelopmental context [80, 81].

## Conclusions

We discover here that a *dfmr1*^*50M*^ stock has accumulated genetic background mutations spread across 8 QTLs, which promote supernumerary synapse projections in the well-mapped giant fiber escape circuit. Excess axonal projections are present from the early stages of synaptogenesis, later incorporate mixed chemical and electrical synapses, and are maintained at maturity. These supernumerary synapses only occur between defined circuit neurons, leading to intra-circuit hyperconnectivity. This suggests that the defect is not in target recognition, but rather in the regulation of synapse number. As synapse formation and refinement are critical processes related to numerous neurodevelopment disorders, including intellectual disability (ID) and autism spectrum disorder (ASD), it is critical to understand underlying molecular mechanisms. Genetic background is known to be a key modifier of disease manifestation in ID/ASD states. Candidate genes identified here via a combination of bulked segregant analysis (BSA) and whole genome sequencing (WGS) provide the means to interrogate new molecular mechanisms driving intra-circuit hyperconnectivity within these disease conditions, with the goal of generating therapeutic intervention strategies in the future.

## Methods

### Drosophila genetics

All animals were maintained on a standard cornmeal/agar/molasses food in a 12-h light to dark cycling incubator at 25 °C. Timed egg lays were collected for 2–3 days, and experimental animals were selected from rearing tubes 10–14 days later, unless otherwise noted. Please see Additional File 1 for the *Drosophila* lines used in genetic crosses. Genetic constructs were recombined as needed for the experiments described below. Genotype was confirmed by visible markers, or PCR when necessary.

### Dye iontophoresis

Dye injection was performed as previously reported [36, 59]. Briefly; glass electrodes (Kwik-Fil Borosilicate glass 1B100F-4, World Precision Instruments) were pulled on a laser puller (Model P-2000, Sutter Instrument Company) to 10MΩ resistance (3 M KCl). Electrodes were filled with 0.25% TRITC-dextran (10 kDa, Life Technologies) and 7% neurobiotin (Vector Laboratories, RRID:AB_2313575) in ddH_2_O. Filled electrodes were placed on a silver-chloride wire mounted on a PCS-5000 micromanipulator (Burleigh). In physiological saline, animals were cut along the dorsal midline to access the cervical connective, where electrodes were inserted into the GFI axon [120]. A square-pulse stimulator (Grass S48, Astro-Med) provided 7.5100 ms pulses/s (2 min, 20 nA injected current) monitored by an AxoClamp2B amplifier. A Digidata data acquisition system (1320A, Axon Instruments) was controlled with Clampex 9.2 software.

### Confocal imaging

Brains were fixed in 4% paraformaldehyde/sucrose (Electron Microscopy Services) in phosphate-buffered saline (PBS, pH 7.2, Life Technology) for 30 min, washed 3X with PBS, and then blocked for 1 h with 1% bovine serum albumin (BSA, Sigma-Aldrich) in PBST (PBS + 0.2% Triton X-100; Thermo Fisher Scientific). Primary and secondary labeling was performed for either 2 h at room temperature (RT) or overnight at 4 °C. All probes were diluted in PBST with 0.2% BSA. The following probes were used: Streptavidin::Cy5 (1:20, SA1011 Thermo Fisher), rabbit anti-ShakB (1:200, [58]), rabbit anti-GFP (1:2000; ab290, Abcam, RRID:AB_303395), FITC Goat anti-GFP (1:500; ab6662, Abcam, RRID:AB_305635), Rabbit anti-RFP (1:500; 600-401-379, Rockland, RRID:AB_2209751), Alexa 488-conjugated donkey anti-goat (1:250; A-11055, Thermo Fisher, RRID:AB_2534102), Alexa 488-conjugated donkey anti-rabbit (1:250; R37118, Thermo Fisher, RRID:AB_2556546), Alexa 568-conjugated donkey anti-rabbit (1:250; A10042, Thermo Fisher, RRID:AB_2534017), Alexa 647-conjugated donkey anti-rabbit (1:250; A-31573, Thermo Fisher, RRID:AB_2536183), and Alexa 633-conjugated goat anti-rabbit (1:250; A-21071, Thermo Fisher, RRID:AB_141419). Preparations were then washed 3X for 30 min in PBST, 1X in PBS, and mounted on glass microscope slides (Probe On Plus 25 × 75 × 1.0 mm, Thermo Fisher Scientific) in 2, 2′-Thiodiethanol (TDE, Sigma-Aldrich [121]). To prevent crushing, double-sided poster tape (Scotch) was placed on each side of the brains. Coverslips (No. 1.5H, Zeiss) were sealed with nail polish (Hard as Nails, Sally Hansen). Fluorescent images were collected using either a Zeiss LSM 510 META confocal microscope or a ZEISS LSM 880 confocal microscope with an Airyscan module, as indicated in the figure legends. Images show maximum Z-stack projections, unless otherwise noted in the figure legends. Occasional misaligned bidirectional scans were corrected using the FIJI plugin “Correct X Shift.”

### Western blotting

Brains were dissected from adult females in PBS with a protease inhibitor (cOmplete mini EDTA-free protease inhibitor cocktail; Roche). Four brains were collected in RIPA buffer (150 mM NaCl, 1% Triton X-100, 50 mM Tris, 0.5% Sodium deoxycholate, 0.1% SDS, 1 mM EDTA, 50 mM TRIS, 1 mM PMSF, Protease Inhibitor Cocktail; Sigma-Aldrich) on ice and sonicated for 20 s (Branson Model 102C, Sonifier 250 microtip). Samples were mixed with 4X LDS buffer (ThermoFisher) with 5% beta-mercaptoethanol (Sigma-Aldrich), incubated at RT for 20 min, boiled at 100 °C for 10 min, and centrifuged at 14,000 RPM for 10 min. Two brain protein equivalents were loaded on a 4%–16% Bis Tris SDS gel (ThermoFisher) in 1x MES buffer (ThermoFisher). Protein was transferred overnight in 1X transfer buffer/20% methanol (ThermoFisher). The membrane was dried for 1 h, blocked with 2% milk (Kroger) in TBS-T (150 mM NaCl, 0.1% Tween, 5 mM KCl, 25 mM Tris, pH 7.6) for 1 h at RT, and then stained with primary antibodies in 2% milk/TBS-T for 2 h at RT. The antibodies used were mouse anti-FMRP (1:3000, Sigma-Aldrich F4554) and rabbit anti-α-tubulin (1:40,000, AbCam Ab52866). Membranes were washed (6X 5-min TBS-T) and then incubated in secondary antibodies in 2% milk/TBS-T for 2 h at RT. The antibodies used were 800 Goat anti-mouse (1:20,000, Rockland) and Alexa 680 goat anti-rabbit (1:20,000, ThermoFisher). Membranes were again washed (6X 5-min TBS-T), and then imaged (LI-COR Odyssey).

### Whole genome sequencing and bulked segregant analysis

Bulked segregant lines were created with multiple generations of inbreeding. To begin, *w*^*1118*^ males were crossed with *w*^*1118*^; *dfmr1*^*50M*^*/*TM6B, *tb*, *hu*, *gfp* females, and the transheterozygous offspring (*w*^*1118*^; *dfmr1*^*50M*^/+) were interbred. The offspring were then interbred for a total of 12 generations. Animals from the 9th, 10th, 11th, and 12th generations were GFI-injected using the above dye iontophoresis method for projection quantification, with the body cryopreserved at − 80 °C. Bodies were then combined into 3 pools based on quantified GFI projection number; 0–3 projections were placed in the control pool, 5–6 projections in the intermediate pool, and 7+ projections in the strong phenotype pool. The low and high pool limits were designed based *w*^*1118*^ and *dfmr1*^*50M*^ projection profiles. Aggregating all dye-injected *w*^*1118*^ animals from our experiments (*n* = 57) showed 58% had 3 or fewer projections, with a minimum of 0, a mode of 3 (*n* = 17), and a maximum of 6. For *dfmr1*^*50M*^ (*n* = 43), 63% had 7 or more projections with a minimum value of 2, a mode of 9 (*n* = 8), and a maximum of 15. By using 3 projections as our cutoff for the low pool, we captured the modal *w*^*1118*^ point, while minimizing the chance of capturing a *dfmr1*^*50M*^ animal. Based on our aggregated data, selecting animals with 0–3 projections yields 94.3% *w*^*1118*^ and 5.7% *dfmr1*^*50M*^. Similarly, by using 7 as the cutoff for the high pool, we expect to see 100% *dfmr1*^*50M*^ animals and capture the modal value of 9. DNA was extracted from each pool using the Qiagen DNeasy Blood and Tissue extraction kit (Qiagen, Cat # 69504). 150bp paired-end read whole genome sequencing was performed on each of the samples, as well as the *w*^*1118*^ and *dfmr1*^*50M*/50M^ parental lines (Hudson Alpha, Illumina NovaSeq).

### Data analyses

Image analysis was done with FIJI software (version 2, RRID:SCR_002285 [122, 123]). GFI projection numbers were from one GFI bend, below the IB. If both GFI arms were visible, the projection number was averaged. GFI projection lengths were quantified using the FIJI Simple Neurite Trace plugin and were only included if their total length was greater than or equal to 2 μm [124]. For all branched GFI projections, the longest continuous branch was followed and the whole structure was counted as a single projection. Projection quantification was not performed in a blinded manner. For anti-ShakB fluorescence quantification, the TRITC dye injection signal was used to create the region of interest (ROI) encompassing the GFI bend. This ROI was then overlaid onto the ShakB channel, and number and total area of punctae above a background threshold (65) were automatically summed from a maximum z projection image using the FIJI “Analyze Particles” tool [122]. For BSA analysis, samples were aligned using SpeedSeq using Flybase *Drosophila* reference genome build 6.28 [125]. BAM files were deposited with SRA under BioProjectID: PRJNA625647. The Samtools depth function was used to calculate depth [126]. For the allele frequency/difference analyses and MULTIPOOL, variants were first called using the Samtools mpileup function and the bcftools call function and processed/plotted with the Pandas, Numpy, and Matplotlib packages in Python2.7 ([75, 126], J. Wang, personal communication). For QTLseqr and PyBSASeq analyses, variants were called and filtered to vcf files using GATK HaplotypeCaller, CombineGVCFs, and GenotypeGVCFs functions, followed by VariantsToTable to create a delimited file [127, 128]. For QTLseqr analyses, the data were analyzed in R using the recommended workflow. For PyBSASeq, data were analyzed in Python3.6 with the recommended settings and an alpha (ltaSNP threshold) of *p* < 0.001 [76, 77, 129]. The Y chromosome was excluded from tests, as the synaptic phenotype was equivalent in males and females. All analysis was performed using dmel-all-chromosome-r6.28.fasta as the reference sequence [130].

### Statistics

All statistical analyses were performed using Prism software (GraphPad, version7, RRID:SCR_002798). All single pairwise comparisons were performed using two-tailed student’s *t* tests. Multiple comparisons were performed using an unpaired one-way ANOVA, with Tukey–Kramer pairwise post hoc tests. In all figures, graphs show the mean ± SEM with the statistical comparisons displayed as: NS (not significant; *p* > 0.05), *p* < 0.05 (*), *p* < 0.01 (**) and *p* < 0.001 (***).

## Supplementary information

**Additional file 1: Table S1.** List of *Drosophila* stocks used in this study. Stocks used in this study are listed by full genotype, Bloomington stock identification number, a brief description of purpose in study, and a reference. Numerous lines were recombined for the purpose of this study and do not have an associated reference.

**Additional file 2: Table S2.** List of protein coding genes and miRNAs in the 8 QTLs. Protein coding genes and miRNAs are listed on separate tabs for each QTL associated with the synaptic projection phenotype. Each tab contains the QTL chromosome and range. The genes are listed in order of their coordinates, with strand indicated by + or -.

## Data Availability

All data generated or analyzed during this study are included in this published article, its supplementary information files and publicly available repositories. The sequencing dataset supporting the conclusions of this article is available in the SRA repository under SRA accession: PRJNA625647. https://www.ncbi.nlm.nih.gov/bioproject/PRJNA625647/
